# Supplementary material for: T cell mediated immunity against influenza H5N1 nucleoprotein, matrix and hemagglutinin derived epitopes in H5N1 survivors and non-H5N1 subjects
Source: PeerJ. 2021 Mar 10;9:e11021. doi: 10.7717/peerj.11021 (PMC7955671; doi:10.7717/peerj.11021)
Supplement: Supplemental Information 1 [file peerj-09-11021-s001.docx]

**Supplementary information**

**Human subjects and detection of neutralizing antibodies against influenza A viruses**

This study comprised two groups of subjects: H5N1 survivors and non-H5N1 subjects. An ELISA-based microNT assay was employed for detecting the presence of neutralizing antibodies against H1N1, H3N2 and HPAI H5N1 viruses. Previous our work reported that all of four H5N1 survivors had markedly high antibody titers against H5N1 virus (*Kitphati et al., 2009*). In addition, they also contained neutralizing antibodies to seasonal H1N1 and H3N2 viruses as shown in Table S1A. In contrast, the non-H5N1 subjects (10 patients infected with H3N2 virus and 23 healthy individuals) had no neutralizing antibody against H5N1 virus as screen at the serum dilution of 1:10; while they possessed antibodies directed against H1N1 and/or H3N2 viruses as shown in Table S1B.

**Reference**

**Kitphati R, Pooruk P, Lerdsamran H, Poosuwan S, Louisirirotchanakul S, Auewarakul P, Chokphaibulkit K, Noisumdaeng P, Sawanpanyalert P, Puthavathana P. 2009.** Kinetics and longevity of antibody response to influenza A H5N1 virus infection in humans. *Clinical and Vaccine Immunology* **16(7)**:978-81 DOI 10.1128/CVI.00062-09.

**Table S1A.** Demographic data, time at blood specimen collection and neutralizing antibodies against H5N1, H1N1 and H3N2 viruses of the four H5N1 survivors

| **Survivor no.** | **Age (years)** | **Gender** | **Blood samples** | **Time at blood collection after disease onset** | **NT Ab titer to** | | |
| --- | --- | --- | --- | --- | --- | --- | --- |
|  |  |  |  |  | **H5N1** | **H3N2** | **H1N1** |
| 1 | 32 | F | 1 | 1y 6m | 160 | ND | ND |
|  |  |  | 2 | 2y 6m | 160 | 10 | 640 |
|  |  |  | 3 | 3y 3m | 80 | ND | ND |
|  |  |  | 4 | 3y 8m | 80 | ND | ND |
|  |  |  | 5 | 4y 2m | 80 | ND | ND |
| 2 | 29 | M | 1 | 2y 2m | 160 | ND | ND |
|  |  |  | 2 | 3y 2m | 160 | 80 | 20 |
|  |  |  | 3 | 3y 10m | 160 | ND | ND |
|  |  |  | 4 | 4y 3m | 80 | ND | ND |
| 3 | 7 | M | 1 | 20d | 640 | ND | ND |
|  |  |  | 2 | 11m | 80 | <10 | 320 |
|  |  |  | 3 | 1y 5m | 40 | ND | ND |
|  |  |  | 4 | 2y 2m | 40 | ND | ND |
|  |  |  | 5 | 2y 7m | 40 | ND | ND |
|  |  |  | 6 | 3y 1m | 40 | ND | ND |
| 4 | 2 | M | 1 | 2y 3m | 80 | ND | ND |
|  |  |  | 2 | 3y 3m | 80 | ND | ND |
|  |  |  | 3 | 3y 11m | 80 | <10 | 160 |
|  |  |  | 4 | 4y 5m | 40 | ND | ND |
|  |  |  | 5 | 4y 11m | 40 | ND | ND |

F; female, M; male, y; year, m; month, d; day, NT Ab; neutralizing antibody, ND; not determine

H5N1: A/Thailand/1(KAN-1)/2004 (H5N1) virus, H3N2: A/Siriraj ICRC/SI-154/2008 (H3N2), an A/Brisbane/10/2007 (H3N2)-like virus, H1N1: A/Thailand/Siriraj-Rama-TT/2004 (H1N1), an A/New Caledonia/20/1999 (H1N1)-like virus

**Table S1B.** Neutralizing antibodies against influenza A viruses in non-H5N1 subjects

| **Subject no.** | **Gender** | **Age (years)** | **Status** | **Blood samples** | **NT antibody to** | | |
| --- | --- | --- | --- | --- | --- | --- | --- |
|  |  |  |  |  | **H5N1** | **H3N2** | **H1N1** |
| 1 | F | 28 | H3N2 patient | Convalescent blood | <10 | 80 | 160 |
| 2 | F | 24 | H3N2 patient | Acute blood | <10 | 80 | 320 |
|  |  |  |  | Convalescent blood | <10 | 640 | 320 |
| 3 | F | 25 | H3N2 patient | Acute blood | <10 | 80 | 320 |
|  |  |  |  | Convalescent blood | <10 | 640 | 320 |
| 4 | F | 27 | H3N2 patient | Acute blood | <10 | 80 | 160 |
|  |  |  |  | Convalescent blood | <10 | 320 | 160 |
| 5 | F | 41 | H3N2 patient | Acute blood | <10 | 640 | 640 |
|  |  |  |  | Convalescent blood | <10 | 640 | 1280 |
| 6 | F | 36 | H3N2 patient | Acute blood | <10 | 80 | 80 |
|  |  |  |  | Convalescent blood | <10 | 40 | 160 |
| 7 | F | 27 | H3N2 patient | Acute blood | <10 | 160 | 80 |
|  |  |  |  | Convalescent blood | <10 | 1280 | 80 |
| 8 | F | 49 | H3N2 patient | Acute blood | <10 | 640 | 160 |
|  |  |  |  | Convalescent blood | <10 | 640 | 320 |
| 9 | F | 24 | H3N2 patient | Acute blood | <10 | 160 | 5120 |
|  |  |  |  | Convalescent blood | <10 | 5120 | 5120 |
| 10 | F | 27 | H3N2 patient | Acute blood | <10 | 160 | 20 |
|  |  |  |  | Convalescent blood | <10 | 80 | 40 |
| 11 | M | 39 | Healthy | Single blood | <10 | 160 | 640 |
| 12 | F | 27 | Healthy | Single blood | <10 | 80 | 1280 |
| 13 | F | 29 | Healthy | Single blood | <10 | 320 | 1280 |
| 14 | M | 27 | Healthy | Single blood | <10 | 80 | 2560 |

**Table S1B (continued).** Neutralizing antibodies against influenza A viruses in non-H5N1 subjects

| **Subject no.** | **Gender** | **Age (years)** | **Status** | **Blood samples** | **NT Ab titer to** | | |
| --- | --- | --- | --- | --- | --- | --- | --- |
|  |  |  |  |  | **H5N1** | **H3N2** | **H1N1** |
| 15 | F | 41 | Healthy | Single blood | <10 | 20 | 1280 |
| 16 | F | 51 | Healthy | Single blood | <10 | 80 | 320 |
| 17 | F | 25 | Healthy | Single blood | <10 | 1280 | 1280 |
| 18 | F | 43 | Healthy | Single blood | <10 | 160 | 320 |
| 19 | F | 43 | Healthy | Single blood | <10 | 80 | 80 |
| 20 | F | 56 | Healthy | Single blood | <10 | 1280 | 80 |
| 21 | F | 51 | Healthy | Single blood | <10 | 320 | 10240 |
| 22 | F | 46 | Healthy | Single blood | <10 | 160 | 80 |
| 23 | F | 23 | Healthy | Single blood | <10 | 640 | 640 |
| 24 | M | 25 | Healthy | Single blood | <10 | 160 | 640 |
| 25 | F | 28 | Healthy | Single blood | <10 | 160 | 160 |
| 26 | F | 24 | Healthy | Single blood | <10 | 20 | 640 |
| 27 | M | 26 | Healthy | Single blood | <10 | 10 | 5120 |
| 28 | F | 25 | Healthy | Single blood | <10 | 160 | 320 |
| 29 | F | 25 | Healthy | Single blood | <10 | 20 | 640 |
| 30 | F | 24 | Healthy | Single blood | <10 | 40 | 640 |
| 31 | M | 26 | Healthy | Single blood | <10 | 320 | 640 |
| 32 | F | 23 | Healthy | Single blood | <10 | 160 | 320 |
| 33 | F | 23 | Healthy | Single blood | <10 | 160 | 80 |

F; female, M; male, NT Ab; neutralizing antibody

H5N1: A/Thailand/1(KAN-1)/2004 (H5N1) virus, H3N2: A/Siriraj ICRC/SI-154/2008 (H3N2), an A/Brisbane/10/2007 (H3N2)-like virus, H1N1: A/Thailand/Siriraj-Rama-TT/2004 (H1N1), an A/New Caledonia/20/1999 (H1N1)-like virus
