# Supplementary material for: T cell mediated immunity against influenza H5N1 nucleoprotein, matrix and hemagglutinin derived epitopes in H5N1 survivors and non-H5N1 subjects
Source: PeerJ. 2021 Mar 10;9:e11021. doi: 10.7717/peerj.11021 (PMC7955671; doi:10.7717/peerj.11021)
Supplement: Supplemental Information 2 [file peerj-09-11021-s002.docx]

**Table S2.** Percentages of [amino acid sequence](https://www.sciencedirect.com/topics/medicine-and-dentistry/peptide-sequence) identities of NP, HA, NA, M1 and M2 among H5N1 viruses isolated from various hosts during 2004 to 2008 in Thailand.

| No. | Virus name | Host | Clade | Amino acid identities to KAN-1 virus (clade 1) | | | | |
| --- | --- | --- | --- | --- | --- | --- | --- | --- |
|  |  |  |  | NP | HA | NA | M1 | M2 |
| 1 | A/Thailand/16/2004 | Human | 1 | 100 | 99.4 | 99.5 | 99.6 | 100 |
| 2 | A/Thailand/2(SP-33)/2004 | Human | 1 | 100 | 99.2 | 99.5 | 99.6 | 100 |
| 3 | A/Thailand/5(KK-494)/2004 | Human | 1 | 99.7 | 99.8 | 99.7 | 98.8 | 100 |
| 4 | A/Thailand/676/2005 | Human | 1 | 99.5 | 98.9 | 98.6 | 99.6 | 100 |
| 5 | A/Thailand/NBL1/2006 | Human | 1 | 100 | 98.7 | 94.9 | 100 | 98.9 |
| 6 | A/chicken/Kalasin/NIAH316/2004 | Chicken | 1 | 100 | 99.4 | 98.6 | 100 | 97.9 |
| 7 | A/chicken/NaraThiwat/NIAH1703/2004 | Chicken | 1 | 100 | 99.6 | 99.1 | 98.8 | 97.9 |
| 8 | A/chicken/Kohn Kaen/NIAH330/2004 | Chicken | 1 | 99.7 | 99.4 | 100 | 99.6 | 100 |
| 9 | A/chicken/Suphanburi/1/2004 | Chicken | 1 | 100 | 99.4 | 100 | 99.6 | 100 |
| 10 | A/chicken/Suphanburi/NIAH7540/2004 | Chicken | 1 | 99.7 | 99.4 | 99.3 | 99.6 | 100 |
| 11 | A/chicken/Suphanburi/NIAH108192/2005 | Chicken | 1 | 99.7 | 99.4 | 98 | 99.6 | 100 |
| 12 | A/chicken/Phichit/NIAH606988/2006 | Chicken | 1 | 99.7 | 99.2 | 99.1 | 99.2 | 100 |
| 13 | A/chicken/Thailand/TS02/2006 | Chicken | 1 | 100 | 99.8 | 100 | 99.2 | 100 |
| 14 | A/chicken/Thailand/TS03/2006 | Chicken | 1 | 99.7 | 99.2 | 98.4 | 99.2 | 100 |
| 15 | A/chicken/Sukhothai/NIAH114843/2008 | Chicken | 1 | 100 | 98.9 | 97.3 | 98.8 | 98.9 |
| 16 | A/chicken/Thailand/ICRC-V586/2008 | Chicken | 1 | 99.7 | 98.9 | 96.6 | 98.8 | 97.9 |
| 17 | A/chicken/Thailand/ST-351/2008 | Chicken | 1 | 100 | 99.4 | 99.3 | 99.6 | 100 |
| 18 | A/chicken/Uthaithani/NIAH115067/2008 | Chicken | 1 | 100 | 98.7 | 98 | 98.8 | 96.9 |
| 19 | A/duck/Ang Thong/71(2)/2004 | Duck | 1 | 99.7 | 99.8 | 100 | 99.6 | 100 |
| 20 | A/duck/Angthong/72/2004 | Duck | 1 | 99.7 | 99.8 | 100 | 99.6 | 100 |
| 21 | A/duck/Angthong/NIAH8246/2004 | Duck | 1 | 99.7 | 99.4 | 98.8 | 99.6 | 100 |
| 22 | A/duck/Thailand/TS01/2006 | Duck | 1 | 100 | 99.8 | 100 | 99.2 | 100 |
| 23 | A/little grebe/Thailand/Phichit-01/2004 | Little grebe | 1 | 100 | 99.6 | 99.7 | 99.6 | 100 |
| 24 | A/open-billed stork/Bangkok/LBD0111F/2004 | Open-billed stork | 1 | 100 | 99.8 | 99.3 | 99.6 | 98.9 |
| 25 | A/open-billed stork/Nakhonsawan/BBD0104F/2004 | Open-billed stork | 1 | 100 | 99.6 | 99.7 | 99.2 | 100 |
| 26 | A/open-billed stork/Suphanburi/TSD0912F/2004 | Open-billed stork | 1 | 100 | 99.6 | 99.7 | 99.6 | 98.9 |
| 27 | A/openbill stork/Thailand/VSMU-4-NSN/2004 | Open-billed stork | 1 | 100 | 99.8 | 99.7 | 99.6 | 100 |
| 28 | A/open-billed stork/Nakhonsawan/BBD3309M/2005 | Open-billed stork | 1 | 99.1 | 99.1 | 98.8 | 99.6 | 98.9 |
| 29 | A/pigeon/Thailand/VSMU-7-NPT/2004 | Pigeon | 1 | 99.7 | 99.6 | 100 | 99.6 | 100 |
| 30 | A/pigeon/Thailand/VSMU-11-KRI/2005 | Pigeon | 1 | 100 | 99.1 | 99.5 | 99.2 | 100 |
| 31 | A/quail/Angthong/71/2004 | Quail | 1 | 100 | 99.8 | 99.5 | 99.6 | 100 |
| 32 | A/quail/Phathumthani/NIAH2711/2004 | Quail | 1 | 100 | 99.8 | 100 | 99.6 | 100 |
| 33 | A/quail/Nakhon Pathom/NIAH7562/2005 | Quail | 1 | 99.7 | 99.4 | 99.1 | 99.6 | 100 |
| 34 | A/tree sparrow/Thailand/VSMU-14-KRI/2005 | Tree sparrow | 1 | 100 | 99.2 | 99.3 | 100 | 98.9 |
| 35 | A/Tiger/Thailand/VSMU-1-SPB/2004 | Tiger | 1 | 100 | 99.8 | 100 | 99.6 | 100 |
