# Supplementary material for: T cell mediated immunity against influenza H5N1 nucleoprotein, matrix and hemagglutinin derived epitopes in H5N1 survivors and non-H5N1 subjects
Source: PeerJ. 2021 Mar 10;9:e11021. doi: 10.7717/peerj.11021 (PMC7955671; doi:10.7717/peerj.11021)
Supplement: Supplemental Information 3 [file peerj-09-11021-s003.docx]

**Supplementary information**

**Overlapping peptide**

1. H5N1-NP overlapping peptides

The NP with 498 amino acids in length (accession no. AAV35112) is encoded from the fifth segment of 1,497 nucleotides (start codon is ATG and stop codon is TAA). There were a total of 49 peptides, each of which has 20 amino acid residues except NP_481-498_, which contains 18 amino acid residues. The peptide sequences are shown in Table S3A.

2. H5N1-M1 and M2 overlapping peptides

The M1 of 252 amino acids in length (accession no. AAV35110) is encoded from the seventh segment of 759 nucleotides (start codon is ATG and stop codon is TGA), whereas M2 which is 97 amino acids long (accession no. AAV35111) is encoded from the spliced RNA of 294 nucleotides in the seventh segment (start codon is ATG and stop codon is TAA). A total of 25 peptides for M1 and 10 peptides for M2 were synthesized. Each peptide contained 20 amino acid residues except the last peptide of M1_241-252_, which contains 12 amino acid residues, and the last peptide of M2_81-97_ which contains 17 amino acids. The peptide sequences are shown in Table S3B.

3. H5N1-HA overlapping peptides

The HA of 568 amino acids in length (accession no. AAS65615) is encoded from the forth segment of 1,708 nucleotides (start codon is ATG and stop codon is TAA). There were a total of 56 peptides, each of which has 20 amino acid residues except HA_551-568_, which contains 18 amino acid residues. The peptide sequences are shown in Table S3C.

These peptides were mixed into various pools using two-dimensional matrix system. Working concentration of 40 µg/ml of individual peptide was present in each peptide pool. There were a total of 14 pools for NP (A1-A7 and B1-B7), 12 pools for both M1 and M2 (A1-A6 and B1-B6) and 15 pools for HA (A1-A8 and B1-B7) (Tables S3D-S3F).

**Table S3A.** Sequences of NP overlapping peptides

| **Protein** | **Position** | **Sequence** | **Position** | **Sequence** |
| --- | --- | --- | --- | --- |
| NP | 1-20 | MASQGTKRSYEQMETGGERQ | 10-30 | EQMETGGERQNATEIRASVG |
|  | 21-40 | NATEIRASVGRMVSGIGRFY | 31-50 | RMVSGIGRFYIQMCTELKLS |
|  | 41-60 | IQMCTELKLSDYEGRLIQNS | 51-70 | DYEGRLIQNSITIERMVLSA |
|  | 61-80 | ITIERMVLSAFDERRNRYLE | 71-90 | FDERRNRYLEEHPSAGKDPK |
|  | 81-100 | EHPSAGKDPKKTGGPIYRRR | 91-110 | KTGGPIYRRRDGKWVRELIL |
|  | 101-120 | DGKWVRELILYDKEEIRRIW | 111-130 | YDKEEIRRIWRQANNGEDAT |
|  | 121-140 | RQANNGEDATAGLTHLMIWH | 131-150 | AGLTHLMIWHSNLNDATYQR |
|  | 141-160 | SNLNDATYQRTRALVRTGMD | 151-170 | TRALVRTGMDPRMCSLMQGS |
|  | 161-180 | PRMCSLMQGSTLPRRSGAAG | 171-190 | TLPRRSGAAGAAVKGVGTMV |
|  | 181-200 | AAVKGVGTMVMELIRMIKRG | 191-210 | MELIRMIKRGINDRNFWRGE |
|  | 201-220 | INDRNFWRGENGRRTRIAYE | 211-230 | NGRRTRIAYERMCNILKGKF |
|  | 221-240 | RMCNILKGKFQTAAQRAMMD | 231-250 | QTAAQRAMMDQVRESRNPGN |
|  | 241-260 | QVRESRNPGNAEIEDLIFLA | 251-270 | AEIEDLIFLARSALILRGSV |
|  | 261-280 | RSALILRGSVAHKSCLPACV | 271-290 | AHKSCLPACVYGLAVASGYD |
|  | 281-300 | YGLAVASGYDFEREGYSLVG | 291-310 | FEREGYSLVGIDPFRLLQNS |
|  | 301-320 | IDPFRLLQNSQVFSLIRPNE | 311-330 | QVFSLIRPNENPAHKSQLVW |
|  | 321-340 | NPAHKSQLVWMACHSAAFED | 331-350 | MACHSAAFEDLRVSSFIRGT |
|  | 341-360 | LRVSSFIRGTRVVPRGQLST | 351-370 | RVVPRGQLSTRGVQIASNEN |
|  | 361-380 | RGVQIASNENMEAMDSNTLE | 371-390 | MEAMDSNTLELRSRYWAIRT |
|  | 381-400 | LRSRYWAIRTRSGGNTNQQR | 391-410 | RSGGNTNQQRASAGQISVQP |
|  | 401-420 | ASAGQISVQPTFSVQRNLPF | 411-430 | TFSVQRNLPFERATIMAAFT |
|  | 421-440 | ERATIMAAFTGNTEGRTSDM | 431-450 | GNTEGRTSDMRTEIIRMMES |
|  | 441-460 | RTEIIRMMESARPEDVSFQG | 451-470 | ARPEDVSFQGRGVFELSDEK |
|  | 461-480 | RGVFELSDEKATNPIVPSFD | 471-490 | ATNPIVPSFDMNNEGSYFFG |
|  | 481-498 | MNNEGSYFFGDNAEEYDN |  |  |

**Table S3B.** Sequences of M1 and M2 overlapping peptides

| **Protein** | **Position** | **Sequence*** | **Position** | **Sequence*** |
| --- | --- | --- | --- | --- |
| M1 | 1-20 | MSLLTEVETYVLSIIPSGPL | 10-30 | VLSIIPSGPLKAEIAQKLED |
|  | 21-40 | KAEIAQKLEDVFAGKNTDLE | 31-50 | VFAGKNTDLEALMEWLKTRP |
|  | 41-60 | ALMEWLKTRPILSPLTKGIL | 51-70 | ILSPLTKGILGFVFTLTVPS |
|  | 61-80 | GFVFTLTVPSERGLQRRRFV | 71-90 | ERGLQRRRFVQNALNGNGDP |
|  | 81-100 | QNALNGNGDPNNMDRAVKLY | 91-110 | NNMDRAVKLYKKLKREITFH |
|  | 101-120 | KKLKREITFHGAKEVALSYS | 111-130 | GAKEVALSYSTGALASCMGL |
|  | 121-140 | TGALASCMGLIYNRMGTVTT | 131-150 | IYNRMGTVTTEVAFGLVCAT |
|  | 141-160 | EVAFGLVCATCEQIADSQHR | 151-170 | CEQIADSQHRSHRQMATTTN |
|  | 161-180 | SHRQMATTTNPLIRHENRMV | 171-190 | PLIRHENRMVLASTTAKAME |
|  | 181-200 | LASTTAKAMEQMAGSSEQAA | 191-210 | QMAGSSEQAAEAMEIANQAR |
|  | 201-220 | EAMEIANQARQMVQAMRTIG | 211-230 | QMVQAMRTIGTHPNSSAGLR |
|  | 221-240 | THPNSSAGLRDNLLENLQAY | 231-250 | DNLLENLQAYQKRMGVQMQR |
|  | 241-252 | QKRMGVQMQRFK |  |  |
| M2 | 1-20 | MSLLTEVETPTRNEWECRCS | 10-30 | TRNEWECRCSDSSDPIVVAA |
|  | 21-40 | DSSDPIVVAANIIGILHLIL | 31-50 | NIIGILHLILWILDRLFFKC |
|  | 41-60 | WILDRLFFKCIYRRLKYGLK | 51-70 | IYRRLKYGLKRGPATAGVPE |
|  | 61-80 | RGPATAGVPESMREEYRQEQ | 71-90 | SMREEYRQEQQSAVDVDDGH |
|  | 81-97 | QSAVDVDDGHFVNIELE | 78-97 | QEQQSAVDVDDGHFVNIELE |

**Table S3C.** Sequences of HA overlapping peptides

| **Protein** | **Position** | **Sequence** | **Position** | **Sequence** |
| --- | --- | --- | --- | --- |
| HA | 1-20 | MEKIVLLFAIVSLVKSDQIC | 10-30 | VSLVKSDQICIGYHANNSTE |
|  | 21-40 | IGYHANNSTEQVDTIMEKNV | 31-50 | QVDTIMEKNVTVTHAQDILE |
|  | 41-60 | TVTHAQDILEKTHNGKLCDL | 51-70 | KTHNGKLCDLDGVKPLILRD |
|  | 61-80 | DGVKPLILRDCSVAGWLLGN | 71-90 | CSVAGWLLGNPMCDEFINVP |
|  | 81-100 | PMCDEFINVPEWSYIVEKAN | 91-110 | EWSYIVEKANPVNDLCYPGD |
|  | 101-120 | PVNDLCYPGDFNDYEELKHL | 111-130 | FNDYEELKHLLSRINHFEKI |
|  | 121-140 | LSRINHFEKIQIIPKSSWSS | 131-150 | QIIPKSSWSSHEASLGVSSA |
|  | 141-160 | HEASLGVSSACPYQRKSSFF | 151-170 | CPYQRKSSFFRNVVWLIKKN |
|  | 161-180 | RNVVWLIKKNSTYPTIKRSY | 171-190 | STYPTIKRSYNNTNQEDLLV |
|  | 181-200 | NNTNQEDLLVLWGIHHPNDA | 191-210 | LWGIHHPNDAAEQTKLYQNP |
|  | 201-220 | AEQTKLYQNPTTYISVGTST | 211-230 | TTYISVGTSTLNQRLVPRIA |
|  | 221-240 | LNQRLVPRIATRSKVNGQSG | 231-250 | TRSKVNGQSGRMEFFWTILK |
|  | 241-260 | RMEFFWTILKPNDAINFESN | 251-270 | PNDAINFESNGNFIAPEYAY |
|  | 261-280 | GNFIAPEYAYKIVKKGDSTI | 271-290 | KIVKKGDSTIMKSELEYGNC |
|  | 281-300 | MKSELEYGNCNTKCQTPMGA | 291-310 | NTKCQTPMGAINSSMPFHNI |
|  | 301-320 | INSSMPFHNIHPLTIGECPK | 311-330 | HPLTIGECPKYVKSNRLVLA |
|  | 321-340 | YVKSNRLVLATGLRNSPQRE | 331-350 | TGLRNSPQRERRRKKRGLFG |
|  | 341-360 | RRRKKRGLFGAIAGFIEGGW | 351-370 | AIAGFIEGGWQGMVDGWYGY |
|  | 361-380 | QGMVDGWYGYHHSNEQGSGY | 371-390 | HHSNEQGSGYAADKESTQKA |
|  | 381-400 | AADKESTQKAIDGVTNKVNS | 391-410 | IDGVTNKVNSIIDKMNTQFE |
|  | 401-420 | IIDKMNTQFEAVGREFNNLE | 411-430 | AVGREFNNLERRIENLNKKM |
|  | 421-440 | RRIENLNKKMEDGFLDVWTY | 431-450 | EDGFLDVWTYNAELLVLMEN |
|  | 441-460 | NAELLVLMENERTLDFHDSN | 451-470 | ERTLDFHDSNVKNLYDKVRL |
|  | 461-480 | VKNLYDKVRLQLRDNAKELG | 471-490 | QLRDNAKELGNGCFEFYHKC |
|  | 481-500 | NGCFEFYHKCDNECMESVRN | 491-510 | DNECMESVRNGTYDYPQYSE |
|  | 501-520 | GTYDYPQYSEEARLKREEIS | 511-530 | EARLKREEISGVKLESIGIY |
|  | 521-540 | GVKLESIGIYQILSIYSTVA | 531-550 | QILSIYSTVASSLALAIMVA |
|  | 541-560 | SSLALAIMVAGLSLWMCSNG | 551-568 | GLSLWMCSNGSLQCRICI |

**Table S3D.** H5N1-NP peptide pools by 2-dimensional matrix system

| **Pool** | **A1** | **A2** | **A3** | **A4** | **A5** | **A6** | **A7** |
| --- | --- | --- | --- | --- | --- | --- | --- |
| **B1** | NP_1-20_ | NP_21-40_ | NP_41-60_ | NP_61-80_ | NP_81-100_ | NP_101-120_ | NP_121-140_ |
| **B2** | NP_141-160_ | NP_161-180_ | NP_181-200_ | NP_201-220_ | NP_221-240_ | NP_241-260_ | NP_261-280_ |
| **B3** | NP_281-300_ | NP_301-320_ | NP_321-340_ | NP_341-360_ | NP_361-380_ | NP_381-400_ | NP_401-420_ |
| **B4** | NP_421-440_ | NP_441-460_ | NP_461-480_ | NP_481-498_ | NP_10-30_ | NP_31-50_ | NP_51-70_ |
| **B5** | NP_71-90_ | NP_91-110_ | NP_111-130_ | NP_131-150_ | NP_151-170_ | NP_171-190_ | NP_191-210_ |
| **B6** | NP_211-230_ | NP_231-250_ | NP_251-270_ | NP_271-290_ | NP_291-310_ | NP_311-330_ | NP_331-350_ |
| **B7** | NP_351-370_ | NP_371-390_ | NP_391-410_ | NP_411-430_ | NP_431-450_ | NP_451-470_ | NP_471-490_ |

**Table S3E.** H5N1-M peptide pools by 2-dimensional matrix system

| **Pool** | **A1** | **A2** | **A3** | **A4** | **A5** | **A6** |
| --- | --- | --- | --- | --- | --- | --- |
| **B1** | M1_1-20_ | M1_21-40_ | M1_41-60_ | M1_61-80_ | M1_81-100_ | M1_101-120_ |
| **B2** | M1_121-140_ | M1_141-160_ | M1_161-180_ | M1_181-200_ | M1_201-220_ | M1_221-240_ |
| **B3** | M1_241-252_ | M1_10-30_ | M1_31-50_ | M1_51-70_ | M1_71-90_ | M1_91-110_ |
| **B4** | M1_111-130_ | M1_131-150_ | M1_151-170_ | M1_171-190_ | M1_191-210_ | M1_211-230_ |
| **B5** | M1_231-250_ | M2_1-20_ | M2_21-40_ | M2_41-60_ | M2_61-80_ | M2_81-97_ |
| **B6** | M2_10-30_ | M2_31-50_ | M2_51-70_ | M2_71-90_ | M2_78-97_ |  |

**Table S3F.** H5N1-HA peptide pools by 2-dimensional matrix system

| **Pool** | **A1** | **A2** | **A3** | **A4** | **A5** | **A6** | **A7** | **A8** |
| --- | --- | --- | --- | --- | --- | --- | --- | --- |
| **B1** | HA_1-20_ | HA_21-40_ | HA_41-60_ | HA_61-80_ | HA_81-100_ | HA_101-120_ | HA_121-140_ | HA_141-160_ |
| **B2** | HA_161-180_ | HA_181-200_ | HA_201-220_ | HA_221-240_ | HA_241-260_ | HA_261-280_ | HA_281-300_ | HA_301-320_ |
| **B3** | HA_321-340_ | HA_341-360_ | HA_361-380_ | HA_381-400_ | HA_401-420_ | HA_421-440_ | HA_441-460_ | HA_461-480_ |
| **B4** | HA_481-500_ | HA_501-520_ | HA_521-540_ | HA_541-560_ | HA_10-30_ | HA_31-50_ | HA_51-70_ | HA_71-90_ |
| **B5** | HA_91-110_ | HA_111-130_ | HA_131-150_ | HA_151-170_ | HA_171-190_ | HA_191-210_ | HA_211-230_ | HA_231-250_ |
| **B6** | HA_251-270_ | HA_271-290_ | HA_291-310_ | HA_311-330_ | HA_331-350_ | HA_351-370_ | HA_371-390_ | HA_391-410_ |
| **B7** | HA_411-430_ | HA_431-450_ | HA_451-470_ | HA_471-490_ | HA_491-510_ | HA_511-530_ | HA_531-550_ | HA_551-568_ |
