# Supplementary material for: T cell mediated immunity against influenza H5N1 nucleoprotein, matrix and hemagglutinin derived epitopes in H5N1 survivors and non-H5N1 subjects
Source: PeerJ. 2021 Mar 10;9:e11021. doi: 10.7717/peerj.11021 (PMC7955671; doi:10.7717/peerj.11021)
Supplement: Supplemental Information 4 [file peerj-09-11021-s004.docx]

**Table S4.** Magnitude and longevity of T cell responses to H5N1 NP, M and HA peptides in H5N1 survivors

| **Survivor no.** | **Time after disease onset** | ***Ex vivo* IFN-γ responses** | | | | | |
| --- | --- | --- | --- | --- | --- | --- | --- |
|  |  | **NP peptide** | **SFCs/10^6^ PBMCs** | **M peptide** | **SFCs/10^6^ PBMCs** | **HA peptide** | **SFCs/10^6^ PBMCs** |
| 1 | 1y 6m | NP_1-20_ | 260 | M1_121-140_ | 150 | ND | ND |
|  |  | NP_111-130_ | 90 | M1_201-220_ | 114 |  |  |
|  | 2y 1m | ND | ND | ND | ND | HA_461-480_ | 133 |
|  | 3y 3m | NP_1-20_ | 259 | M1_121-140_ | 81 | ND | ND |
|  |  | NP_111-130_ | 56 | M1_201-220_ | 83 |  |  |
|  | 3y 8m | NP_1-20_ | 266 | ND | ND | ND | ND |
|  |  | NP_111-130_ | 63 |  |  |  |  |
|  | 4y 2m | NP_1-20_ | 274 | M1_121-140_ | 60 | ND | ND |
|  |  | NP_111-130_ | 51 | M1_201-220_ | 67 |  |  |
| 2 | 2y 2 m | NP_411-430_ | 631 | M1_1-20_ | 95 | ND | ND |
|  |  |  |  | M_91-110_ | 53 |  |  |
|  |  |  |  | M1_241-252_ | 83 |  |  |
|  | 2y 8m | ND | ND | ND | ND | HA_41-60_ | 67 |
|  |  |  |  |  |  | HA_251-270_ | 66 |
|  |  |  |  |  |  | HA_291-310_ | 59 |
|  | 3y 2m | ND | ND | M1_1-20_ | 108 | ND | ND |
|  |  |  |  | M1_91-110_ | 52 |  |  |
|  |  |  |  | M1_241-252_ | 90 |  |  |
|  | 3y 10m | NP_411-430_ | 568 | ND | ND | ND | ND |
|  | 4y 3m | NP_411-430_ | 522 | M1_1-20_ | 84 | ND | ND |
|  |  |  |  | M1_91-110_ | 59 |  |  |
|  |  |  |  | M1_241-252_ | 84 |  |  |
| 3 | 5m | No response | No response | ND | ND | ND | ND |
|  | 11m | No response | No response | ND | ND | ND | ND |
|  | 1y 5m | No response | No response | ND | ND | ND | ND |
|  | 2y 2m | No response | No response | ND | ND | ND | ND |
|  | 2y 7m | No response | No response | ND | ND | ND | ND |
|  | 3y 1m | No response | No response | ND | ND | ND | ND |
| 4 | 3y 3m | No response | No response | ND | ND | ND | ND |

y, year; m, month; ND; not determined; No response means that the numbers of spot forming cells (SFCs) in the ELISpot reaction wells using peptide pools are not greater than 2 times of the negative control

NP_1-20_ MASQGTKRSYEQMETGGERQ NP_111-130_ YDKEEIRRIWRQANNGEDAT

NP_411-430_ TFSVQRNLPFERATIMAAFT M1_1-20_ MSLLTEVETYVLSIIPSGPL

M1_91-110_ NNMDRAVKLYKKLKREITFH M1_121-140_ TGALASCMGLIYNRMGTVTT

M1_201-220_ EAMEIANQARQMVQAMRTIG M1_241-252_ QKRMGVQMQRFK

HA_41-60_ TVTHAQDILEKTHNGKLCDL HA_251-270_  PNDAINFESNGNFIAPEYAY

HA_461-480_  VKNLYDKVRLQLRDNAKELG HA_291-310_ NTKCQTPMGAINSSMPFHNI
