# Supplementary material for: T cell mediated immunity against influenza H5N1 nucleoprotein, matrix and hemagglutinin derived epitopes in H5N1 survivors and non-H5N1 subjects
Source: PeerJ. 2021 Mar 10;9:e11021. doi: 10.7717/peerj.11021 (PMC7955671; doi:10.7717/peerj.11021)
Supplement: Supplemental Information 5 [file peerj-09-11021-s005.docx]

**Table S5.** Monofunction and polyfunction of peptide-specific effector T cell lines

| **T cell subset** | **Expression marker** | **Percentages of effector T cells after stimulation with** | | | |
| --- | --- | --- | --- | --- | --- |
|  |  | **TBCLs without peptide** | **Peptide- pulsed TBCLs** | **rVac-pSC11 infected TBCLs** | **rVac-NP infected TBCLs** |
| Survivor no. 1 | IFN-γ^+^ | 1.73 | 22.66 | 1.84 | 12.89 |
| CD4^+^ T cells | TNF-α^+^ | 1.93 | 23.12 | 1.96 | 15.47 |
|  | CD107a^+^ | 1.73 | 24.97 | 2.45 | 16.19 |
|  | IFN-γ^+^TNF-α^+^ | 0.4 | 8.5 | 0.7 | 11.6 |
|  | IFN-γ^+^CD107a^+^ | 0.19 | 19.42 | 0.61 | 11.03 |
|  | TNF-α^+^CD107a^+^ | 1.35 | 21.73 | 1.23 | 13.9 |
|  | IFN-γ^+^TNF-α^+^CD107a^+^ | 1.35 | 23.58 | 1.35 | 14.18 |
| Survivor no. 1 | IFN-γ^+^ | 4.35 | 15.12 | 3.67 | 69.1 |
| CD8^+^ T cells | TNF-α^+^ | 17.39 | 34.71 | 35.78 | 93.13 |
|  | CD107a^+^ | 13.04 | 36.77 | 36.7 | 93.13 |
|  | IFN-γ^+^TNF-α^+^ | 1.8 | 68.2 | 4.4 | 15.2 |
|  | IFN-γ^+^CD107a^+^ | 0 | 14.78 | 0.9 | 68.24 |
|  | TNF-α^+^CD107a^+^ | 13.04 | 34.36 | 35.78 | 93.13 |
|  | IFN-γ^+^TNF-α^+^CD107a^+^ | 13.04 | 34.36 | 35.78 | 93.13 |
| Survivor no. 2 | IFN-γ^+^ | 9.5 | 88.91 | 11.03 | 59.6 |
| CD4^+^ T cells | TNF-α^+^ | 9.5 | 88.12 | 13.74 | 54.3 |
|  | CD107a^+^ | 7.47 | 14.92 | 11.21 | 13.81 |
|  | IFN-γ^+^TNF-α^+^ | 5.0 | 84.1 | 6.3 | 50.2 |
|  | IFN-γ^+^CD107a^+^ | 2.71 | 12.97 | 5.06 | 13.73 |
|  | TNF-α^+^CD107a^+^ | 4.75 | 14.22 | 7.78 | 12.51 |
|  | IFN-γ^+^TNF-α^+^CD107a^+^ | 5.43 | 15.47 | 9.95 | 15.81 |
| Survivor no. 2 | IFN-γ^+^ | 2.62 | 67.98 | 1.53 | 67.78 |
| CD8^+^ T cells | TNF-α^+^ | 4.9 | 64.58 | 3.84 | 51.42 |
|  | CD107a^+^ | 3.89 | 38.35 | 3.71 | 40.65 |
|  | IFN-γ^+^TNF-α^+^ | 2.3 | 63.4 | 1.2 | 53.4 |
|  | IFN-γ^+^CD107a^+^ | 1.07 | 34.89 | 0.96 | 36.36 |
|  | TNF-α^+^CD107a^+^ | 3.69 | 35.91 | 3.33 | 34.81 |
|  | IFN-γ^+^TNF-α^+^CD107a^+^ | 3.69 | 37.76 | 3.33 | 39.6 |
