# Supplementary figures and images for: T cell mediated immunity against influenza H5N1 nucleoprotein, matrix and hemagglutinin derived epitopes in H5N1 survivors and non-H5N1 subjects
Source: PeerJ. 2021 Mar 10;9:e11021. doi: 10.7717/peerj.11021 (PMC7955671; doi:10.7717/peerj.11021)

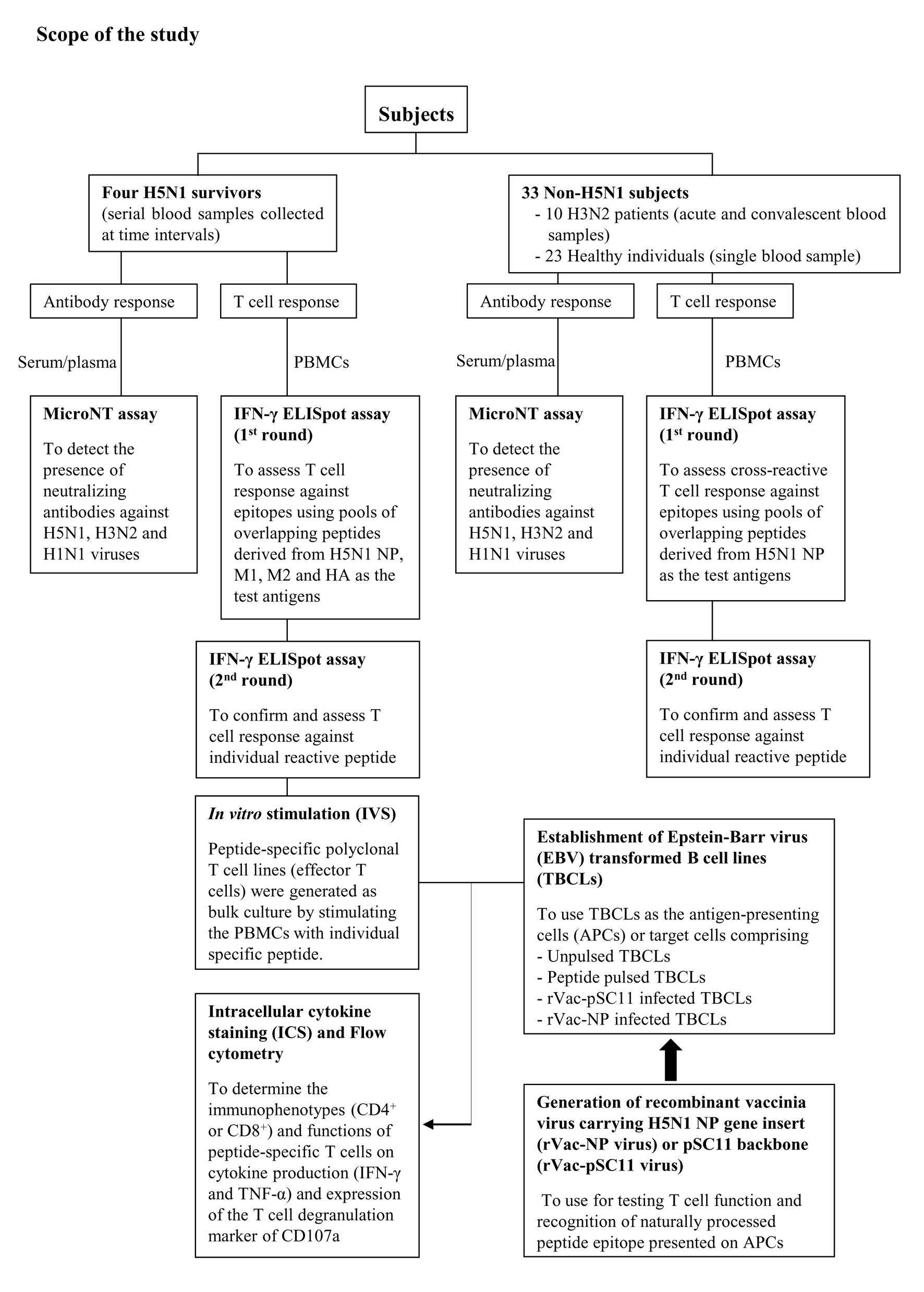

Supplement: Supplemental Information 6 [file peerj-09-11021-s006.png]

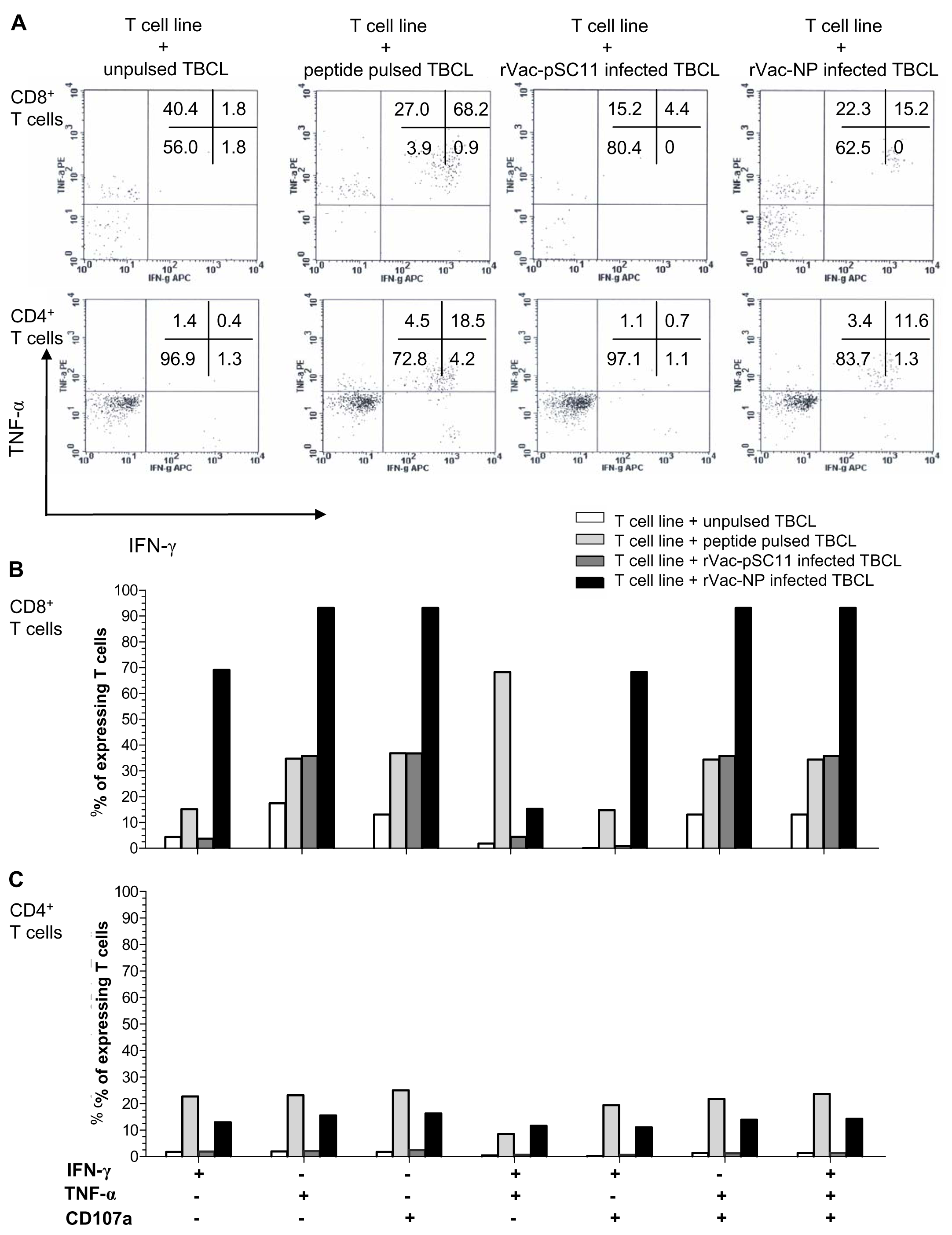

Supplement: Supplemental Information 7 — The T cell function which singly or simultaneously produced IFN-γ and TNF-α (A) and/or upregulation of CD107a degranulation marker (B) in survivor no.1. The percentages of both specific CD4+ and CD8+ T cells that expressed IFN-γ+, TNF-α+ and/or CD107a markedly increased in recognition of the target cells pulsed with peptide or infected with rVac-NP virus. [file peerj-09-11021-s007.png]
